# Reducing the rate and duration of Re-ADMISsions among patients with unipolar disorder and bipolar disorder using smartphone-based monitoring and treatment – the RADMIS trials: study protocol for two randomized controlled trials

**DOI:** 10.1186/s13063-017-2015-3

**Published:** 2017-06-15

**Authors:** Maria Faurholt-Jepsen, Mads Frost, Klaus Martiny, Nanna Tuxen, Nicole Rosenberg, Jonas Busk, Ole Winther, Jakob Eyvind Bardram, Lars Vedel Kessing

**Affiliations:** 1 Psychiatric Center Copenhagen, Rigshospitalet, Blegdamsvej 9, DK-2100 Copenhagen, Denmark; 2 Psychiatric Center Copenhagen, Rigshospitalet, University Hospitalet of Copenhagen, Copenhagen, Denmark; 3 IT University of Copenhagen, Copenhagen, Denmark; 4 Department of Applied Mathematics and Computer Science, Technical University of Denmark, Lyngby, Denmark; 5 Copenhagen Center for Health Technology, Technical University of Denmark, Copenhagen, Denmark

## Abstract

**Background:**

Unipolar and bipolar disorder combined account for nearly half of all morbidity and mortality due to mental and substance use disorders, and burden society with the highest health care costs of all psychiatric and neurological disorders. Among these, costs due to psychiatric hospitalization are a major burden. Smartphones comprise an innovative and unique platform for the monitoring and treatment of depression and mania. No prior trial has investigated whether the use of a smartphone-based system can prevent re-admission among patients discharged from hospital. The present RADMIS trials aim to investigate whether using a smartphone-based monitoring and treatment system, including an integrated clinical feedback loop, reduces the rate and duration of re-admissions more than standard treatment in unipolar disorder and bipolar disorder.

**Methods:**

The RADMIS trials use a randomized controlled, single-blind, parallel-group design. Patients with unipolar disorder and patients with bipolar disorder are invited to participate in each trial when discharged from psychiatric hospitals in The Capital Region of Denmark following an affective episode and randomized to either (1) a smartphone-based monitoring system including (a) an integrated feedback loop between patients and clinicians and (b) context-aware cognitive behavioral therapy (CBT) modules (intervention group) or (2) standard treatment (control group) for a 6-month trial period. The trial started in May 2017. The outcomes are (1) number and duration of re-admissions (primary), (2) severity of depressive and manic (only for patients with bipolar disorder) symptoms; psychosocial functioning; number of affective episodes (secondary), and (3) perceived stress, quality of life, self-rated depressive symptoms, self-rated manic symptoms (only for patients with bipolar disorder), recovery, empowerment, adherence to medication, wellbeing, ruminations, worrying, and satisfaction (tertiary). A total of 400 patients (200 patients with unipolar disorder and 200 patients with bipolar disorder) will be included in the RADMIS trials.

**Discussion:**

If the smartphone-based monitoring system proves effective in reducing the rate and duration of re-admissions, there will be basis for using a system of this kind in the treatment of unipolar and bipolar disorder in general and on a larger scale.

**Trial registration:**

ClinicalTrials.gov, ID: NCT03033420. Registered 13 January 2017. Ethical approval has been obtained.

**Electronic supplementary material:**

The online version of this article (doi:10.1186/s13063-017-2015-3) contains supplementary material, which is available to authorized users.

## Background

Unipolar disorder and bipolar disorder are common mental diseases with a lifetime prevalence of 15–20% and 1–2%, respectively [1, 2]. Unipolar disorder and bipolar disorder combined account for nearly half of all morbidity and mortality due to mental and substance use disorders [3] and burden society with the highest health care costs of all psychiatric and neurological disorders [4].

Although psychiatric treatment internationally, and in Denmark, has shifted more from inpatient treatment to outpatient treatment during recent decades, costs due to psychiatric hospitalization are still a major burden comprising two thirds of all direct costs in the five Regions of Denmark [5]. Patients with affective disorders are more frequently hospitalized than any other patient group, counting more than 10,800 patients in 2013 among the entire Danish population of 5.4 million people, and 20% of all psychiatric hospitalizations in Denmark [6]. The cost of these hospitalizations alone is estimated to be DKK648 million (approximately €8.7 million) per year in Denmark (10,800 hospitalizations with a mean of 20 days’ stay for a cost of 3000 DKK/day (€400)). The discharge period after hospitalization is a high-risk period with a more than 300 times increased risk of suicide during the first week [7] and a high risk of re-admission [8, 9]. Patients are often afraid of leaving the hospital, feeling alone with the prospect of an appointment with a physician 1 or more months ahead. A pilot study from our group in which 45 patients discharged from psychiatric hospital self-monitored symptoms using a daily computer-based self-monitoring system emphasized the pivotal importance of continued contact with a clinician such as a nurse [10]. Smartphones comprise a unique platform for the monitoring and treatment of depression and mania. Depression and mania are associated with changes in several behavioral components (e.g., reduction in activity level, change in speech) and motivational states (e.g., anhedonia), some of which may be detectable using readily available smartphone sensors [11–13]. Prior work done by our group has developed and tested a unique smartphone-based system for the treatment of bipolar disorder (the MONARCA system) [14, 15]. The MONARCA system is capable of collecting subjective self-assessment data and automatically generated objective smartphone data from patients on a daily basis while allowing simple bidirectional communication between clinicians and the patients [15]. We find it likely that daily contact with a psychiatric hospital nurse using a bidirectional feedback system combined with objective smartphone-based early warning signs, assessed using a smartphone app, will reduce the risk of re-admission and the risk of relapse of depressive and (hypo)manic symptoms.

Treatment of unipolar disorder and bipolar disorder applies a variety of methods, including antidepressants, mood stabilizers, psychoeducation, and cognitive behavioral psychotherapy (CBT).

CBT has proven efficacious in the treatment and prevention of depressive episodes in unipolar disorder [16], but the effect in relation to bipolar disorder is controversial [17]. Depressive symptoms dominate bipolar disorder as 80% of episodes are depressive [18], but bipolar depression is difficult to treat as only a few drugs have proven effects [19] and psychological interventions have proven ambient effects on depressive symptoms revealing positive effects in some studies [20–22], but not in others [23–25]. Specifically targeting depressive rumination, rumination-focused cognitive therapy and concreteness training (a facilitated self-help intervention intended to increase specificity of processing in patients with depression) have shown encouraging results in the treatment of residual depression [26]. However, the number of CBT therapists is very low compared with the huge demand for CBT. Internet- and computer-based CBT systems exist and have been proved effective in relation to depression [27]. Recently, CBT has been suggested for smartphones [28] but the effect has never been tested in a randomized controlled trial (RCT).

In the RADMIS trial we will develop a smartphone-based CBT program that includes methods to relieve depressive ruminations [23, 29]. The smartphone-based system used in the RADMIS trials is inspired by the MONARCA system (see below), and is called the Monsenso system. By using the sensing, computational and communication capabilities of smartphones, it is possible to continuously monitor an individual’s context including physical activity, location, and environment. Thus, smartphones hold significant promise as a platform to monitor behavioral and environmental indicators of depression and mania and for treatment, facilitating early intervention and smartphone-based CBT.

Prior research on using smartphones in the detection of unipolar disorder and bipolar disorder has shown promising but preliminary results. A small study on eight patients with depression (not including a control group) found that sensor data from smartphones could detect social patterns [30]. Another study found that sensors from smartphones were effective at detecting social and sleep behaviors among patients with depression [31].

The MONARCA system, developed and tested by our group [14, 15], has been proved highly usable and useful by patients with bipolar disorder with a high self-assessment adherence (87–95%), and was considered to help patients to better manage their disease [32]. A study investigating the effect of the MONARCA system (daily self-monitoring including a two-level feedback loop to clinicians) in a RCT found no overall effect in primary analyses but secondary analyses suggested that the system was able to reduce manic symptoms, but may sustain depressive symptoms in patients with bipolar disorder [33]. These results are in accordance with the findings that psychological interventions for bipolar disorder in general have more effect on manic than depressive symptoms and more effect on preventing that treating acute depression [23–25]. Consequently, emphasis on depressive symptoms should be a high priority including considerations on mechanisms to reduce the negative processing bias and depressive ruminations in patients with unipolar disorder and bipolar disorder [23, 33].

Finally, research by our group found that automatically generated objective smartphone data reflect illness activity in patients with bipolar disorder. Data on physical activity (the number of changes in cell tower ID/day), social activity (the number and duration of phone calls/day and the number of text messages/day) [11–13] and voice features collected during phone calls [34] correlated significantly with the severity of clinically rated depressive and manic symptoms. Although these findings are encouraging and innovative, there is a need to integrate self-monitored smartphone data with automatically generated objective smartphone data on physical and social activity, as well as speech and sleep, into a composite smartphone-generated electronic measure. This composite measure should be modeled to have a high correlation with depressive and manic symptoms, and a high predictive ability to identify upcoming affective episodes for the individual patient in order to facilitate early intervention.

### Hypotheses

Using a smartphone-based monitoring and treatment system for daily electronic self-monitoring, including an integrated feedback loop, on both subjective and automatically generated behavioral data on measures of illness activity (phone usage, social activity, physical activity, mobility, voice recognition and sleep) and context-aware CBT modules, the Monsenso system, reduces the rate and duration of re-admission more than standard treatment in adult patients with unipolar disorder or bipolar disorder, respectively, being discharged from hospital.

### Objectives

To investigate in two individual, randomized controlled, single-blind, parallel-group trials whether the use of a smartphone-based monitoring and treatment system, including an integrated feedback loop, on both subjective and automatically generated behavioral data on measures of illness activity (phone usage, social activity, physical activity, mobility, voice recognition and sleep) and context-aware CBT modules, the Monsenso system, reduces the rate and duration of re-admissions more than treatment-as-usual in adult patients with unipolar disorder or bipolar disorder, respectively.

Further, to investigate if the Monsenso system reduces the severity of clinically rated affective symptoms and the number of affective episodes, improves psychosocial functioning, quality of life, severity of self-assessed affective symptoms, recovery, empowerment, adherence to medication, wellbeing, and satisfaction with care, and reduces perceived stress, rumination and worrying more than standard treatment without a smartphone-based system in adult patients with unipolar disorder or bipolar disorder, respectively.

Data will be collected and analyzed separately according to psychiatric diagnosis (unipolar disorder and bipolar disorder).

## Methods

**Fig. 1:**
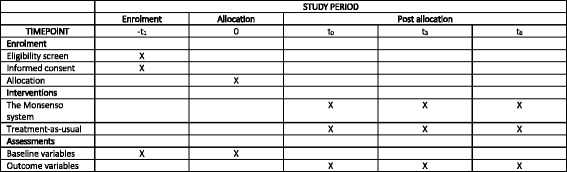
Schedule of enrolment, interventions, and outcome assessments in the RADMIS trials

The present trial protocol is reported according to the CONsolidated Standards Of Reporting Trials (CONSORT) Statement and Standard Protocol Items: Recommendations for Interventional Trials (SPIRIT) [35–37] (Fig. 1, Additional file 1).

The trial protocol describes two individual, randomized controlled, single-blind, parallel-group trials, the RADMIS trials, investigating the effect of using the Monsenso system on a daily basis, this including an integrated feedback loop and context-aware CBT modules, compared with standard treatment in adult patients with unipolar disorder and bipolar disorder, respectively.

### Trial design and study organization

**Fig. 2:**
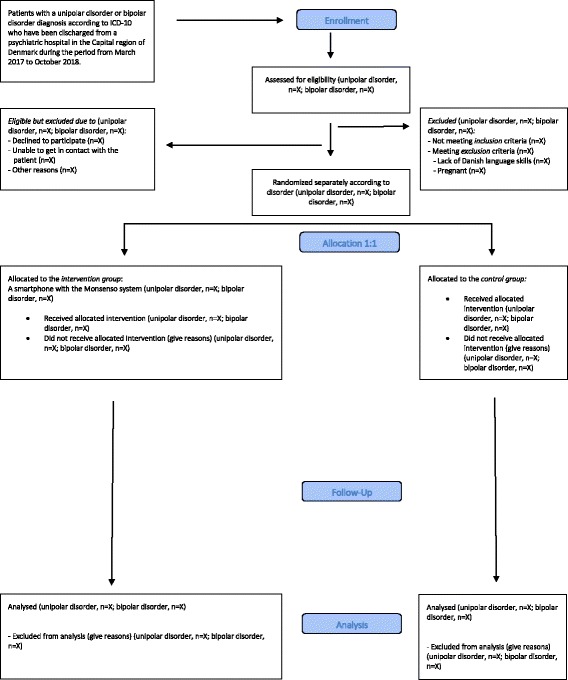
Flow diagram of the RADMIS trials

The RADMIS trials are randomized controlled, single-blind, parallel-group trials with a balanced allocation ratio (1:1) of adult patients with unipolar disorder or bipolar disorder stratified according to the psychiatric center from where the patients are discharged and the number of former hospitalizations (three or less or more than three). The included patients are randomized separately according to psychiatric diagnosis (unipolar disorder or bipolar disorder) to either active use of the Monsenso system (intervention group) or to standard treatment (control group). Thus, the RADMIS trials consists of two separate RCTs with two patient groups investigating the effect of the same intervention (the Monsenso system). The flow diagram of the RADMIS trials is presented in Fig. 2. The trails are conducted at the Psychiatric Center Copenhagen, Rigshospitalet, Copenhagen, Denmark. No changes in study design or methods have been made after trial commencement.

### Participants and settings

Inclusion criteria: all patients over the age of 18 years with a unipolar disorder or bipolar disorder diagnosis, according to the *International Classification of Diseases, version 10* (ICD-10) using Schedules for Clinical Assessments in Neuropsychiatry (SCAN) [38], who are discharged from a psychiatric hospital in The Capital Region of Denmark during the period March 2017 to October 2018 following an affective episode (depression or mania) are invited to participate in the RADMIS trials. This corresponds to approximately 1200 patients with unipolar disorder and 600 patients with bipolar disorder per year. The mental health services in The Capital Region of Denmark covers a recruitment area of the Capital Region, Denmark, corresponding to 1.4 million people.

Exclusion criteria: patients who are pregnant and those who lack Danish language skills are excluded, since these factors may influence the possible effect of the RADMIS intervention.

### Study procedure

Potential participants are invited to participate in the RADMIS trials by contact with the staff during hospitalization. All potential participants who accept to meet with the RADMIS staff for further trial information are screened by trained researcher to make sure that they fulfil the criteria for participation, and are then included in the RADMIS trials. Following inclusion, baseline assessments are performed on all patients, and after these assessments the numbered opaque allocation envelopes are distributed by a research secretary (HGN) to the RADMIS study nurses. The included patients are randomized separately according to psychiatric diagnosis (unipolar disorder or bipolar disorder) to either the intervention group or the control group for a 6-month trial period.

An overview of the outcome assessments conducted by researchers blinded to intervention group is presented in Table 1. Information regarding the primary outcome, re-admissions and duration of re-admissions, will be obtained after completion of the two RCTs by linkage of the unique personal identification number (CPR) which is assigned to all 5.4 million persons living in Denmark [39] with the Danish Psychiatric Central Register [40].

**Table 1:** Outcome assessments during the RADMIS trial

	SCAN and background information	Rating scales	Questionnaires	Clinical information
Baseline	x	x	x	x
Randomization to (1) smartphone-based monitoring and treatment (the intervention group) or (2) treatment-as-usual (the control group)				
3-month follow-up		x	x	x
6-month follow-up		x	x	x

*SCAN* Schedules for Clinical Assessment in Neuropsychiatry interview

Rating scales: Hamilton Depression Rating Scale 17-item (HDRS-17); Young Mania Rating Scale (only for patients with a bipolar disorder diagnosis); Psychosocial functioning test (Functional Assessment Short Test (FAST))

Questionnaires: perceived stress according to Cohen’s Perceived Stress Scale; quality of life according to the WHO Quality of Life-BREF (WHOQOL-BREF); self-rated depressive symptoms according to Beck’s Depressive Inventory (BDI); self-rated depressive symptoms according to the Hamilton Depression Self-rating Scale 6-item (HDRS-6); self-rated manic symptoms according to the Altman Self-rating Scale for Mania (ASRM) (only for patients with a bipolar disorder diagnosis); recovery according to the Recovery Assessment Scale; empowerment according to Rogers’ Empowerment Scale; adherence to medication according to the Medicine Adherence Rating Scale; wellbeing according to the WHO (five) Wellbeing Index; rumination according to the Rumination Response Scale (RRS); worrying according to the Penn State Worry Questionnaire (PSWQ); satisfaction according to the Verona Satisfaction Scale-Affective Disorder (VSS-A). The HDRS-6, the ASRM and the WHO (five) are filled out every month during the study 6-month period

Clinical information: re-admissions and duration of re-admissions (register data); number of affective episodes; number of contacts with clinicians and psychiatric emergency rooms; hospitalizations; medication, etc

### The intervention group

All included patients have been discharged from a psychiatric hospital at The Capital Region of Denmark during the period from May 2017 to October 2018 following an affective episode (depression or mania). All included patients continue standard treatment-as-usual at a community psychiatric centre, a private psychiatrist, a general practitioner or outpatient treatment at a hospital during the trial period.

#### The smartphones

In the RADMIS trials, the Monsenso system is available for smartphones capable of collecting subjective and automatically generated behavioral data on measures of illness activity. All patients randomized to the intervention group are offered the loan of an Android smartphone free of charge for the 6-month trial period. The patients in the intervention group are encouraged to use the Monsenso system for daily electronic self-monitoring. Economic costs from data traffic due to the RADMIS trials are refunded to all participants regardless the choice of smartphone type.

##### Subjective (self-monitored) measures of illness activity in the intervention group

The patients randomized to the intervention group, regardless the choice of smartphone are, on a daily basis, prompted by an alarm in the Monsenso system at a self-chosen time during the day to evaluate subjective measures of illness activity.

The following subjective (self-monitored) measures of illness activity are available for daily evaluation for patients with unipolar disorder as well as bipolar disorder: sleep duration (number of hours slept per night, measured in half-hour intervals), time of falling asleep the previous evening (hh:mm), time woken up the following morning (hh:mm), medicine intake (taken as prescribed/taken with changes (if changes, the patients are asked to specify these)/not taken), anxiety (scored from “not present,” “present to some degree” or “present” on a scale from 0, 1, 2), cognitive problems (scored from “not present,” “present to some degree” or “present” on a scale from 0, 1, 2), alcohol consumption (number of units consumed per day, 0 to +10 scale), stress (scored from “not present,” “present to some degree” or “present” on a scale from 0, 1, 2), menstruation for women (yes/no), individual early warning signs (yes/no), a number (unlimited) of personal parameters (created by the patients themselves), and a free-text note.

Suicidal thoughts will not be evaluated as part of the self-monitoring due to ethical concerns and since deterioration, leading to suicidal thoughts, will be reflected on other of the self-monitored parameters in the system. Suicidal patients will be treated using standard care and taken care of by the usual clinician.

In addition to these items, the following self-monitored measures of illness activity are available for daily evaluation *specifically* for patients with unipolar disorder: mood (scored from “euthymic” to “depressive” on a scale from 0, −0.5, −1, −2, −3), activity level (scored from “normal” to “very low” on a scale from 0, −1, −2, −3), irritability (scored from “not present,” “present to some degree” or “present” on a scale from 0, 1, 2). Similarly, the following additional self-monitored measures of illness activity are available for daily evaluation *specifically* for patients with bipolar disorder: mood (scored from “depressive” to “manic” on a scale from −3, −2, −1, −0.5, 0, +0.5, +1, +2, +3), activity level (scored from “very low” to “very high” on a scale from −3, −2, −1, 0, +1, +2, +3), mixed mood (yes/no), irritability (scored from “not present,” “present to some degree” or “present” on a scale from 0, 1, 2).

**Fig. 3:**
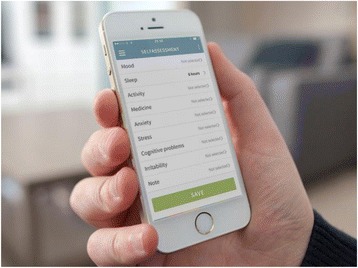
The Monsenso self-assessment system. Screenshot of the smartphone-based self-assessment

**Fig. 4:**
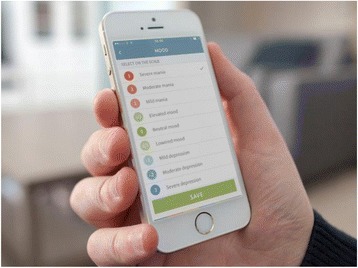
The Monsenso self-assessment system. Screenshot of the smartphone-based self-assessment of mood

After midnight, the entered subjective (self-monitored) measures of illness activity are “locked” and further changes cannot be made. If the patients wish to change their subjective evaluation they can enter a second evaluation in addition to the initial one, and both of the subjective evaluations are then visible for the patient and the health care provider when logging on to in the Monsenso system. If the patients forget to evaluate the subjective measures it is possible to enter and evaluate retrospectively for up to 2 days. It is then noted in the Monsenso system that the subjective measures are collected retrospectively. Screenshots from the Monsenso system are presented in Figs. 3 and 4. A user’s guide for the Monsenso system was developed and handed out to all patients in the intervention group (can be obtained by contacting the corresponding author).

##### Automatically generated behavioral data (objective data) on measures of illness activity

Smartphones are capable of collecting automatically generated behavioral data on measures of illness activity on a daily basis (objective data) during the 6-month trial period.

Examples of some of the automatically generated behavioral data on measures of illness activity collected by the smartphones: (1) phone usage measured as the number of times and amount of time the smartphones screen are turned on/off, battery usage, ambient light, and “proximity” detection, (2) social activity measured as the number of in- and outgoing phone calls and text messages, the duration of in- and outgoing phone calls, the length of the text messages, and the time of the day when the phone calls and/or text messages are made/sent/received, (3) physical activity measured by the step counter in the smartphones, and (4) mobility based on the location estimation available in the smartphones (which again rely on, e.g., GPD or GSM cell tower information depending on specific circumstances). Furthermore, speech activity is collected by extraction of different voice features during phone calls. Voice feature extraction will take place directly on the smartphones and no recording of the actual speech/conversation will take place. The intention is to synthesize these automatically generated behavioral data into one aggregated objective composite measure.

##### Smartphone-based CBT modules

The smartphone-based CBT modules include four modules: psychoeducation, behavioral activation, cognitive restructuring and rumination-focused CBT. The psychoeducation and mood-monitoring system include strategies for detecting and intervening with early signs of relapse. Patients are encouraged to use the module in an individualized and sequential way according to their condition, affective state, and insight and following guidance from the study nurse if wanted. The behavioral activation module was inspired by Martell et al. (2010) and Lejuez et al. (2001) [41, 42]. It includes activity monitoring and activity scheduling and focus on regulation of sleep and daily routines. Several studies have established the efficacy for behavioral activation for treating depression [43–46] including a few studies of behavioral activation using smartphones; however, not including a control standard treatment arm [47, 48]. Further, regulating patterns of activity and sleep are helpful in reducing both depressive and manic symptoms [43, 49].

The cognitive restructuring includes simple techniques to help patients to identify and modify dysfunctional automatic thoughts. Cognitive restructuring is a key element of CBT and is included in a number of studies that show the efficiency of CBT in the treatment of depression including some studies of Internet-based CBT [50, 51], and a few studies including patients with bipolar disorder [20, 22].

A main purpose of the smartphone-based CBT modules was to help patients to reduce depressive ruminations. Therefore, a specific rumination-focused CBT strategy, based on the theory and practice described by Watkins et al. [25], was included. Rumination-focused CBT has shown encouraging results in the treatment of residual depression [25] and the Monsenso system includes the identification and reduction of unhelpful ruminations.

##### The integrated feedback loop between patients and clinicians

Study nurses with experience with unipolar disorder and bipolar disorder are assigned to the patients allocated to the intervention group of the RADMIS trials. The RADMIS study nurses are responsible for the integrated feedback loop. Patients allocated to the intervention group of the RADMIS trials will have the Monsenso application installed on a smartphone. The smartphone automatically transfers the self-monitored subjective measures and, for some smartphones, also the automatically generated behavioral data on measures of illness activity to servers at the hospital through secure connections (I-suite number RHP-2011-03). By giving informed consent to participate in the RADMIS, the patients allow for the RADMIS study nurses and their health care provider to access the monitored data through a secure web interface. The RADMIS study nurses go through the collected data approximately two to three times a week, or more often on patients where it is deemed necessary. A personal homepage is set up on a server allowing the patients to access all their own data through a similar secure web interface.

1. The feedback loop on subjective measures: regardless the type of smartphone a feedback loop on the subjective measures is established. A standard of scoring thresholds for when the RADMIS study nurses initially should react was made. For example, the RADMIS study nurses react if the patients register ≥ −2 on the mood item for 2 days or more, or if the patients register changes in their sleep patterns of 1 h or more for more than 3 days. Following a run-in phase of approximately 2 to 4 weeks of self-monitoring, the patients and the RADMIS study nurses individualize the thresholds for when reaction should be made. Also the RADMIS study nurses and the patients agree on a concordance status in (a) the patients’ most important items for identifying prodromal symptoms of depression as well as (hypo)mania (only for patients with a bipolar disorder diagnosis), (b) the threshold for future early warning signs, and (c) actions to be taken in case of depression or (hypo)mania (only for patients with a bipolar disorder diagnosis)
2. The integrated feedback loop on subjective and automatically generated behavioral data on measures of illness activity: a feedback loop integrating subjective and automatically generated behavioral data on measures of illness activity is established. The feedback loop integrates both subjective and automatically generated behavioral data on measures of illness activity in a flexible and adjustable model (a learning system) resulting in prediction analyses of the collected data providing messages for both the patients and the RADMIS study nurses such as: “You are invited to contact your RADMIS study nurse.”

Actions taken by the RADMIS study nurses, as part of the feedback loop in the intervention group, in case of signs of deterioration of a patient in the intervention group:

1. Contact the patient and give advice on how to handle the situation
2. If the first action is not enough, the study nurses ask the patient to contact the usual physician or other clinician
3. If the above actions are not enough, or if contact with the patient is not possible, the study nurses contact the patient’s usual physician or other clinician themselves
4. In case of acute deterioration and/or severe symptoms, the study nurses recommend the patient to contact the psychiatric emergency service in Copenhagen, Denmark

The Monsenso system was designed in an interactive process between patients with unipolar disorder, bipolar disorder, IT researchers, clinicians, and clinical researchers before the beginning of the RADMIS trial.

### The control group

Patients allocated to the control group continue with their standard treatment-as-usual and are asked to continue communicating as usual using their mobile phone (smartphone).

### Assessments

Researchers blinded to the patients’ allocation of intervention group (MFJ and one other physician) who are not involved in the treatment of the patients carry out all outcome assessments. The unipolar disorder or bipolar disorder diagnoses according to ICD-10 are confirmed by a SCAN interview before inclusion of the patients. The patients are, regardless of randomization group, enrolled for a 6-month trial period and invited for outcome assessments by researchers blinded to intervention at baseline, after 3 months and after 6 months (Table 1).

At each visit with the researchers, the assessments include the following:

1. Clinician-administrated rating scales: the severity of depressive and manic symptoms (manic symptoms only assessed for patients with a bipolar disorder diagnosis) is measured using the Hamilton Depression Rating Scale 17-item (HDRS-17) [52] and the Young Mania Rating Scale (YMRS) [34], respectively, and psychosocial functioning is measured using the Functioning Assessment Short Test (FAST) [53]

2. Questionnaires: perceived stress according to Cohen’s Perceived Stress Scale [54], quality of life according to the WHO Quality of Life-BREF (WHOQOL-BREF) [55], self-rated depressive symptoms according to Beck’s Depressive Inventory (BDI) [56–58], self-rated depressive symptoms according to the Hamilton Depression Self-rating Scale 6-item (HDRS-6) [59], self-rated manic symptoms according to Altman Self-rating Scale for Mania (ASRM) (only for patients with a bipolar disorder diagnosis) [34], recovery according to the Recovery Assessment Scale [60], empowerment according to Rogers’ Empowerment Scale [61], adherence to medication according to the Medicine Adherence Rating Scale [62], wellbeing according to the WHO (five) Wellbeing Index [63], rumination according to the Ruminative Response Scale (RRS), worrying according to the Penn State Worry Questionnaire (PSWQ) [64], and satisfaction according to the Verona Satisfaction Scale-Affective Disorder (VSS-A) [65]. The patients are asked to fill out HDRS-6, ASRM (only for patients with a bipolar diagnosis) and WHO (five) every month during the 6-month trial period in both groups and in both trials.

### Outcomes

#### Primary outcomes

- Rate and duration of hospital re-admissions of patients with unipolar disorder and bipolar disorder, respectively, as according to data retrieved from the Danish Psychiatric Central Register [40]

#### Secondary outcomes

- Severity of depressive symptoms and manic symptoms (manic symptoms only for patients with a bipolar disorder diagnosis) measured using the HDRS-17 and the YMRS, respectively
- Psychosocial functioning according to the Functional Assessment Short Test (FAST)
- Number of depressive episodes and manic episodes (manic episodes only for patients with a bipolar disorder diagnosis) defined as HDRS-17 > 13 and YMRS >13, respectively
- Automatically generated behavioral data measured as an aggregated composite measure

#### Tertiary outcomes

- Perceived stress according to Cohen’s Perceived Stress Scale [54], quality of life according to the WHO Quality of Life-BREF (WHOQOL-BREF) [55], self-rated depressive symptoms according to Beck’s Depressive Inventory (BDI) [56–60], self-rated depressive symptoms according to the Hamilton Depression Self-rating Scale 6-item (HDRS-6) [59], self-rated manic symptoms according to the Altman Self-rating scale for Mania (ASRM) (only for patients with a bipolar disorder diagnosis) [34], recovery according to the Recovery Assessment Scale [60], empowerment according to Roger’s Empowerment Scale [61], adherence to medication according to the Medicine Adherence Rating Scale [62], wellbeing according to the WHO (five) Wellbeing Index [63], rumination according to the Ruminative Response Scale (RRS), worrying according to the Penn State Worry Questionnaire (PSWQ) [64], and satisfaction according to the Verona Satisfaction Scale-Affective Disorder (VSS-A) [65]

No changes in trial outcomes have been made after trial commencement.

### Statistical power and sample size calculation

The statistical power and sample size was calculated using http://stat.ubc.ca/~rollin/stats/ssize/n2.html.

The primary outcomes are differences in the number and duration of re-admissions to a psychiatric hospital between the intervention group and the control group − analyzed separately according to psychiatric diagnosis (unipolar disorder or bipolar disorder). Power calculations are similar for the two RCTs/patient groups (unipolar disorder or bipolar disorder). Based on prior findings aiming to reduce re-hospitalization due to recurrent depression [66] and bipolar disorder [9], combined with presumptions of positive effects of the immediate early contact with patients during the vulnerable period following discharge from a psychiatric hospital, regardless of diagnosis, we expect a reduction in re-admissions from 30% to 15% in the intervention group using smartphone-based treatment. Thus, anticipating a hazard ratio (HR) of 0.50 in the comparison of the intervention group with the control group on the primary outcome, a two-sided risk of type 1 error, *α* of 0.05, a type 2 error risk, *β*, a statistical power of 80%, the sample size (*N*) is calculated as *N* = 200 (100 patients in each arm of each RCT) per RCT and, thus, a total of 400 patients (200 patients with unipolar disorder and 200 patients with bipolar disorder) is estimated to be required for the RADMIS trial. Cumulated durations of re-hospitalizations comprise similar power calculations and sample sizes. Regarding the secondary outcomes, the clinically relevant difference in depressive or manic symptoms is defined as a minimum of three scores on the HDRS-17 or on the YMRS, respectively, and the standard deviation (SD) is set at 7 with a mean score of 12 versus 15 in the intervention group and the control group, respectively. The statistical power to detect a three-score difference in the areas under the curves between the intervention and the control groups on the HDRS-17 or the YMRS, respectively, is 80% with *α* = 0.05 for a two-sample comparison of means including a total of 200 patients (100 patients in each arm) in each of the two RADMIS RCTs.

### Randomization

#### Sequence generation

A computer-generated list of random allocation numbers using Pharma Consulting Group (http://www.pharmaconsultinggroup.com) will be generated. Patients included in the trial are randomized with a balanced allocation ratio of 1:1 to (1) using the Monsenso system, including the integrated feedback loop, based on a combination of subjective self-monitored measures and automatically generated behavioral data on measures of illness activity) including context-aware CBT modules (the intervention group) or to (2) standard treatment-as-usual (the control group) (Fig. 1).

Since the RADMIS trial is single-blinded, random block sizes are used to help preserve unpredictability [67, 68]. The RADMIS study nurses are unaware of the range of numbers in the block sizes.

The study will use a stratified design, where patients are stratified according to the psychiatric centres where patients are discharged from and according to the number of previous hospitalizations (three or less or more than three). The statistical analyses will adjusted for the two stratification variables, and also age and sex as possible prognostic variables. Further, in analyses on continuous variables, potential differences in baseline score on the outcome in question will be included as a potential confounder. If there are no statistically significant main effects of age and sex, these variables will be excluded from the final statistical analyses.

#### Allocation concealment and implementation

The allocation sequence is concealed from the researchers (e.g., MFJ) enrolling and assessing the patients and from the RADMIS study nurses. Allocation is concealed in numbered, opaque and sealed envelopes.

### Blinding

Owing to the type of intervention in the RADMIS trials, the patient, the patients’ health care provider and the RADMIS study nurses are aware of the allocated randomization group. The researchers responsible for outcome assessments, data entry, data analyses, interpretation of analyses and writing of papers are kept blinded to allocation at all times during the trial period, data analyses and interpretation of analyses. The trials are, therefore, single-blinded. The RADMIS study nurses do not collect any outcome measures. At each visit with the researchers, all patients are thoroughly instructed not to mention anything about randomization allocation. In case of unblinding of the included patients’ allocation status, one of the other researchers blinded to intervention in the RADMIS trials will conduct the outcome assessments.

### Statistical methods

Data from all randomized patients are collected until dropout or the end of the trial period. Analysis will be carried out with an intention-to-treat (ITT) approach. The primary outcomes are differences in time to re-admission and duration of re-admissions during the 6-month trial period between the intervention group and the control group. Time to the first re-admission will be estimated using a Kaplan-Meier plot with reasons for censoring being date of death or end of study. The differences in cumulated prevention of re-admission in the intervention group and the control group will tested using a log-rank test. Analysis of secondary and tertiary outcomes will be done employing a linear mixed-effects model with random intercept for each participant and a fixed effect of visit. Differences in outcomes between the intervention group and the control group will be analyzed, firstly in an unadjusted model (except for differences in baseline values of the outcome variable in analyses on continuous variables) and then in models adjusted for the two stratification variables (1) psychiatric center and (2) the number of prior hospitalizations, and also for age and sex as possible prognostic variables. If there are no statistically significant main effects of age and sex, these variables will be excluded from the final analyses. Furthermore, subanalyses will be done employing a linear mixed-effects model with random intercept for each participant and a fixed effect of visit on differences in the HDRS-17 and the YMRS in patients with the presence of depressive and manic symptoms (defined as HDRS-17 > 0 or YMRS >0, respectively) at a given time point during the trial period between the intervention group and the control group. Additionally, in addition to analyses of the secondary outcomes, we will analyze differences in depressive and manic episodes defined as HDRS ≥14 and YMRS ≥14, respectively, during the trial period. Potential interactions between randomization group and visit number on any specific outcome variable in the analyses will be investigated and reported accordingly. The statistical threshold for significance is *p* ≤ 0.05 (two-tailed). Data will be managed by MFJ and entered using Epidata®. All analyses will be done using SPSS, version 22.0 (IBM, New York, NY, USA) and STATA version 12 (StataCorp LP, College Station, TX, USA).

## Discussion

Monitoring of illness activity using smartphones seems promising as an intervention in unipolar disorder and bipolar disorder, but few studies using rigorous methodology have been published. No study investigating the effect of a smartphone-based intervention on rate and duration of re-admissions in both unipolar disorder and bipolar disorder has been published. The RADMIS trials aim to investigate the effect of a smartphone-based monitoring and treatment system on the risk of psychiatric hospitalization, clinical function, and wellbeing in randomized, controlled trials with clearly defined clinical outcome measures.

### Advantages

First, the RADMIS trials’ use a pragmatic design with few exclusion criteria and the results of the trials will be generalizable to patients who are going to be discharged from psychiatric hospitals in general with a diagnosis of unipolar disorder or bipolar disorder and have clinical relevance. The patients are recruited at a critical time point at discharge from hospital at which they still suffer from some affective symptoms and have a need for continued treatment. Following hospitalization, patients often experience a service gap between inpatient and outpatient services, and the RADMIS smartphone-based monitoring and treatment system is developed among others to fill out this gap.

Second, it is a major advantage that clinical information on the primary outcome will be available for all included patients (100%), regardless of whether they drop out of the RADMIS trials or not, as data on date of re-admission and duration of re-hospitalizations routinely are reported nationwide to the Danish Psychiatric Central Register [40]. Also, the information on re-admissions and duration of re-hospitalizations are collected without the risk of unblinding of the researcher and not based on the patients’ subjective evaluations.

Third, information on the secondary outcome measures are based on standardized clinical rating scales often used as the “gold standard,” and are conducted by trained researchers blinded to intervention groups. In contrast to this, the tertiary outcomes are based on the patients’ subjective and unblinded evaluations and are, thus, at risk of performance bias.

Fourth, the intervention used in the RADMIS trials is specifically designed to address the needs of the patients when being discharged from a psychiatric hospitalization. During the design phase of the trial the software was designed in a close collaboration between patients with unipolar disorder and bipolar disorder, IT researchers, clinicians, and clinical researchers during an interactive process.

Fifth, automatically generated behavioral data collected by the smartphones will be combined in an aggregated combined measure of illness activity that will be reported as an additional outcome measure. These data are objective and have been shown to correlate with the severity of depressive and manic symptoms in studies by our group. However, using these data as outcome measures is still experimental and will be developed further during the RADMIS trials. Consequently, the aggregated smartphone-based measure of illness activity is not included as a predefined outcome measure in this study protocol.

Sixth, the Monsenso system will be available for both Android and iPhones. Thus, patients are not excluded due to preference of smartphone type and/or operating system. Patients randomized to the intervention group are offered to loan a smartphone free of charge during the RADMIS trials and, thus, there are no requirements for smartphone access.

### Limitations

#### The intervention group

The RADMIS trials are designed to investigate the effect of the entire Monsenso system, including self-monitoring, automatically generated behavioral data, integrated feedback loop and context-aware CBT modules. Thus, we will not be able to distinguish the effects of the individual components of the intervention.

However, in subanalyses it will be investigated whether there are differences in outcome measures between patients who have used the CBT modules during the trial period and patients who have not.

#### The control group

It is always difficult to define a proper control group. In a previously trial conducted by the authors, an effect of a “placebo smartphone” was most likely not found [32]. Further, during the last years few of the patients participating in our studies have expressed a wish to loan a smartphone free of charge. Thus, we chose not to offer a placebo smartphone in the present RADMIS trial.

#### Secondary and tertiary outcome measures

The secondary outcome measures are differences in clinically rated depressive and manic symptoms as assessed with standardized rating scales. Since the patients are aware of their allocation status these outcome measures are single blinded. The tertiary outcomes are questionnaires evaluated by the patients themselves and are, therefore, unblinded.

### Perspectives

If the Monsenso system proves effective in reducing the rate and duration of re-admissions in patients with unipolar disorder and bipolar disorder in the present trial, there will be basis for using a system of this kind in psychiatric treatment in general and on a larger scale.

### Trial status

The trial is ongoing. Recruitment begins March 2017.

## Additional file

Additional file 1: SPIRIT checklist for the RADMIS trial. (DOC 115 kb)

## Abbreviations

ASRM: The Altman Self-rating Scale for Mania
BDI: Beck’s Depressive Inventory
CBT: Cognitive behavioral psychotherapy
FAST: The Functional Assessment Short Test
HDRS-17: The Hamilton Depression Rating Scale 17-item
HDRS-6: The Hamilton Depression Self-rating Scale 6-item
HR: Hazard ratio
ITT: Intention-to-treat
PSWQ: The Penn State Worry Questionnaire
RCT: Randomized controlled trial
RRS: The Ruminative Response Scale
SCAN: Schedules for Clinical Assessments in Neuropsychiatry
The MONARCA system: Smartphone-based monitoring system for patients with bipolar disorder
The Monsenso system: Smartphone-based monitoring system including CBT modules for patients with both unipolar disorder and bipolar disorder (inspired by the MONARCA system)
The RADMIS trial: Reducing the rate and duration of Re-ADMISsions among patients with unipolar disorder and bipolar disorder using smartphone-based monitoring and treatment
VSS-A: The Verona Satisfaction Scale-Affective Disorder
WHOQOL-BREF: The WHO Quality of Life-BREF
YMRS: The Young Mania Rating Scale

## References

[1] Merikangas KR, Akiskal HS, Angst J, Greenberg PE, Hirschfeld RMA, Petukhova M (2007). Lifetime and 12-month prevalence of bipolar spectrum disorder in the National Comorbidity Survey Replication. Arch Gen Psychiatry.

[2] Kessler RC, Berglund P, Demler O (2003). The epidemiology of major depressive disorder: results from the national comorbidity survey replication (ncs-r). JAMA.

[3] Whiteford HA, Degenhardt L, Rehm J, Baxter AJ, Ferrari AJ, Erskine HE (2013). Global burden of disease attributable to mental and substance use disorders: findings from the Global Burden of Disease Study 2010. Lancet.

[4] Olesen J, Gustavsson A, Svensson M, Wittchen H-U, Jönsson B (2012). on behalf of the CDBE2010 study group, et al. The economic cost of brain disorders in Europe. Eur J Neurol.

[5] Arbejdsgruppe under Regeringens udvalg om Psykiatri. Indsatsen for mennesker med psykiske lidelser- kapacitet, sammenhæng og strktur. Bilagsrapport 1. Afrapportering fra Arbejdsgruppemøde under Regeringens udvalg om Psykiatri. 2013.

[6] The Psychiatric Central Register. [Internet]. 2016. Available from: http://www.kea.au.dk/da/forskning/det-psykiatriske-centralregister.html_2016. Accessed 11 June 2017.

[7] Mortensen PB, Agerbo E, Erikson T, Qin P, Westergaard-Nielsen N (2000). Psychiatric illness and risk factors for suicide in Denmark. Lancet Lond Engl.

[8] Kessing LV, Hansen MG, Andersen PK (2004). Course of illness in depressive and bipolar disorders. Naturalistic study, 1994-1999. Br J Psychiatry.

[9] Kessing LV, Hansen HV, Hvenegaard A, Christensen EM, Dam H, Gluud C (2013). Treatment in a specialised out-patient mood disorder clinic v. standard out-patient treatment in the early course of bipolar disorder: randomised clinical trial. Br J Psychiatry.

[10] Nørregaard L, Løventoft P, Frøkjær E, Lauritsen L, Olsson E, Andersen L, et al. Patient expectations and experiences from a clinical study in psychiatric care using a self-monitoring system. In: NordiCHI’14: Proceedings of the 8th Nordic Conference on Human-Computer Interaction: Fun, Fast, Foundational. Helsinki: Association for Computing Machinery; 2014. p. 991–4.

[11] Faurholt-Jepsen M, Frost M, Vinberg M, Christensen EM, Bardram JE, Kessing LV (2014). Smartphone data as objective measures of bipolar disorder symptoms. Psychiatry Res.

[12] Faurholt-Jepsen M, Vinberg M, Frost M, Christensen EM, Bardram JE, Kessing LV. Smartphone data as an electronic biomarker of illness activity in bipolar disorder. Bipolar Disord. 2015;17(7):715–28. doi:10.1111/bdi.12332. 10.1111/bdi.12332 26395972

[13] Faurholt-Jepsen M. Behavioral activities collected through smartphones and the association with illness activity in bipolar disorder. Int J Methods Psychiatr Res. 2016. In press 10.1002/mpr.1502 PMC6860202 27038019

[14] Bardram J, Frost M, Szanto K, Margu G. The MONARCA self-assessment system: a persuasive personal monitoring system for bipolar patients. In: Proceedings of the 2nd ACM SIGHIT International Health Informatics Symposium (IHI’12) ACM, New York, NY, USA, 21-30. ACM New York, NY, USA; 2012. p. 21–30.

[15] Faurholt-Jepsen M, Vinberg M, Christensen EM, Frost M, Bardram J, Kessing LV. Daily electronic self-monitoring of subjective and objective symptoms in bipolar disorder—the MONARCA trial protocol (MONitoring, treAtment and pRediCtion of bipolAr disorder episodes): a randomised controlled single-blind trial. BMJ Open. 2013;3(7). doi:10.1136/bmjopen-2013-003353. 10.1136/bmjopen-2013-003353 PMC3731717 23883891

[16] Watts SE, Turnell A, Kladnitski N, Newby JM, Andrews G (2015). Treatment-as-usual (TAU) is anything but usual: a meta-analysis of CBT versus TAU for anxiety and depression. J Affect Disord..

[17] Scott J, Paykel E, Morriss R, Bentall R, Kinderman P, Johnson T (2006). Cognitive-behavioural therapy for severe and recurrent bipolar disorders: randomised controlled trial. Br J Psychiatry J Ment Sci..

[18] Judd LL, Akiskal HS, Schettler PJ, Endicott J, Maser J, Solomon DA (2002). The long-term natural history of the weekly symptomatic status of bipolar I disorder. Arch Gen Psychiatry.

[19] Pacchiarotti I, Bond DJ, Baldessarini RJ, Nolen WA, Grunze H, Licht RW (2013). The International Society for Bipolar Disorders (ISBD) task force report on antidepressant use in bipolar disorders. Am J Psychiatry.

[20] Lam DH, Watkins ER, Hayward P, Bright J, Wright K, Kerr N (2003). A randomized controlled study of cognitive therapy for relapse prevention for bipolar affective disorder: outcome of the first year. Arch Gen Psychiatry.

[21] Lam DH, Hayward P, Watkins ER, Wright K, Sham P (2005). Relapse prevention in patients with bipolar disorder: cognitive therapy outcome after 2 years. Am J Psychiatry.

[22] Miklowitz DJ, Otto MW, Frank E, Reilly-Harrington NA, Kogan JN, Sachs GS (2007). Intensive psychosocial intervention enhances functioning in patients with bipolar depression: results from a 9-month randomized controlled trial. Am J Psychiatry.

[23] Perry A, Tarrier N, Morriss R, McCarthy E, Limb K (1999). Randomised controlled trial of efficacy of teaching patients with bipolar disorder to identify early symptoms of relapse and obtain treatment. BMJ.

[24] Simon GE, Ludman EJ, Bauer MS, Unützer J, Operskalski B (2006). Long-term effectiveness and cost of a systematic care program for bipolar disorder. Arch Gen Psychiatry.

[25] Scott J, Colom F. Gaps and limitations of psychological interventions for bipolar disorders. Psychother Psychosom. 2008;77(1):4–11. 10.1159/000110054 18087202

[26] Watkins ER (2009). Depressive rumination: investigating mechanisms to improve cognitive behavioural treatments. Cogn Behav Ther.

[27] Ebert DD, Zarski A-C, Christensen H, Stikkelbroek Y, Cuijpers P, Berking M, et al. Internet and computer-based cognitive behavioral therapy for anxiety and depression in youth: a meta-analysis of randomized controlled outcome trials. PLoS ONE [Internet]. 2015;10(3). Available from: http://www.ncbi.nlm.nih.gov/pmc/articles/PMC4364968/. Accessed on 4 Feb 2016. 10.1371/journal.pone.0119895 PMC4364968 25786025

[28] Ly KH, Janni E, Wrede R, Sedem M, Donker T, Carlbring P (2015). Experiences of a guided smartphone-based behavioral activation therapy for depression: a qualitative study. Internet Interv.

[29] Morriss RK, Faizal MA, Jones AP, Williamson PR, Bolton C, McCarthy JP (2007). Interventions for helping people recognise early signs of recurrence in bipolar disorder. Cochrane Database Syst Rev..

[30] Burns MN, Begale M, Duffecy J, Gergle D, Karr CJ, Giangrande E (2011). Harnessing context sensing to develop a mobile intervention for depression. J Med Internet Res.

[31] Doryab A. Detection of behavior change in people with depression. In: Workshops at the Twenty Eighth AAAI Conference. 2014.

[32] Bardram JE, Frost M, Szánto K, Faurholt-Jepsen M, Vinberg M, Kessing LV. Designing mobile health technology for bipolar disorder: a field trial of the Monarca system. In: Proceedings of the SIGCHI Conference on Human Factors in Computing Systems. CHI: New York; 2013. p. 2627–36.

[33] Faurholt-Jepsen M, Frost M, Ritz C, Christensen EM, Jacoby AS, Mikkelsen RL (2015). Daily electronic self-monitoring in bipolar disorder using smartphones—the MONARCA I trial: a randomized, placebo-controlled, single-blind, parallel group trial. Psychol Med..

[34] Young RC, Biggs JT, Ziegler VE, Meyer DA (1978). A rating scale for mania: reliability, validity and sensitivity. Br J Psychiatry..

[35] Moher D, Hopewell S, Schulz KF, Montori V, Gøtzsche PC, Devereaux PJ (2010). CONSORT 2010 explanation and elaboration: updated guidelines for reporting parallel group randomised trials. BMJ..

[36] Boutron I, Moher D, Altman DG, Schulz KF, Ravaud P (2008). Extending the CONSORT statement to randomized trials of nonpharmacologic treatment: explanation and elaboration. Ann Intern Med.

[37] Chan A-W, Tetzlaff JM, Altman DG, Dickersin K, Moher D (2013). SPIRIT 2013: new guidance for content of clinical trial protocols. Lancet.

[38] Wing JK, Babor T, Brugha T, Burke J, Cooper JE, Giel R (1990). SCAN. Schedules for clinical assessment in neuropsychiatry. Arch Gen Psychiatry.

[39] Malig C (1996). The civil registration system in Denmark.

[40] Munk-Jørgensen P, Mortensen PB (1997). The Danish Psychiatric Central Register. Dan Med Bull.

[41] Martell CR, Dimidjian S, Herman-Dunn R (2010). Behavioral activation for depression: a clinician’s guide.

[42] Lejuez CW, Hopko DR, Hopko SD (2001). A brief behavioral activation treatment for depression. Treatment manual. Behav Modif.

[43] Dimidjian S, Hollon SD, Dobson KS, Schmaling KB, Kohlenberg RJ, Addis ME (2006). Randomized trial of behavioral activation, cognitive therapy, and antidepressant medication in the acute treatment of adults with major depression. J Consult Clin Psychol.

[44] Cuijpers P, van Straten A, Warmerdam L (2007). Behavioral activation treatments of depression: a meta-analysis. Clin Psychol Rev.

[45] Dobson KS, Hollon SD, Dimidjian S, Schmaling KB, Kohlenberg RJ, Gallop R (2008). Randomized trial of behavioral activation, cognitive therapy, and antidepressant medication in the prevention of relapse and recurrence in major depression. J Consult Clin Psychol.

[46] Dimidjian S, BarreraJr M, Martell C, Muñoz RF, Lewinsohn PM (2011). The origins and current status of behavioral activation treatments for depression. Annu Rev Clin Psychol.

[47] Ly KH, Trüschel A, Jarl L, Magnusson S, Windahl T, Johansson R, et al. Behavioural activation versus mindfulness-based guided self-help treatment administered through a smartphone application: a randomised controlled trial. BMJ Open [Internet]. 2014;4(1). Available from: http://www.ncbi.nlm.nih.gov/pmc/articles/PMC3902198/. Accessed 13 June 2016. 10.1136/bmjopen-2013-003440 PMC3902198 24413342

[48] Ly KH, Topooco N, Cederlund H, Wallin A, Bergström J, Molander O, et al. Smartphone-supported versus full behavioural activation for depression: a randomised controlled trial. PLoS ONE [Internet]. 2015;10(5). Available from: http://www.ncbi.nlm.nih.gov/pmc/articles/PMC4444307/. Accessed 13 June 2016. 10.1371/journal.pone.0126559 PMC4444307 26010890

[49] Shen GH, Alloy LB, Abramson LY, Sylvia LG (2008). Social rhythm regularity and the onset of affective episodes in bipolar spectrum individuals. Bipolar Disord.

[50] DeRubeis RJ, Siegle GJ, Hollon SD (2008). Cognitive therapy vs. medications for depression: treatment outcomes and neural mechanisms. Nat Rev Neurosci.

[51] Watts S, Mackenzie A, Thomas C, Griskaitis A, Mewton L, Williams A (2013). CBT for depression: a pilot RCT comparing mobile phone vs. computer. BMC Psychiatry.

[52] Hamilton M (1967). Development of a rating scale for primary depressive illness. Br J Soc Clin Psychol.

[53] Rosa AR, Sánchez-Moreno J, Martínez-Aran A, Salamero M, Torrent C, Reinares M (2007). Validity and reliability of the Functioning Assessment Short Test (FAST) in bipolar disorder. Clin Pract Epidemiol Ment Health CP EMH..

[54] Cohen S, Kamarck T, Mermelstein R (1983). A global measure of perceived stress. J Health Soc Behav.

[55] WHO (1998). Development of the World Health Organization WHOQOL-BREF quality of life assessment. The WHOQOL Group. Psychol Med.

[56] Bech P, Olsen LR (2001). Discovering depression. Ugeskr Laeger.

[57] Bech P, Rasmussen NA, Olsen LR, Noerholm V, Abildgaard W (2001). The sensitivity and specificity of the Major Depression Inventory, using the Present State Examination as the index of diagnostic validity. J Affect Disord.

[58] Olsen LR, Jensen DV, Noerholm V, Martiny K, Bech P (2003). The internal and external validity of the Major Depression Inventory in measuring severity of depressive states. Psychol Med.

[59] Bech P, Gefke M, Lunde M, Lauritzen L, Martiny K. The pharmacopsychometric triangle to illustrate the effectiveness of T-PEMF concomitant with antidepressants in treatment resistant patients: a double-blind, randomised, sham-controlled trial revisited with focus on the patient-reported outcomes. Depress Res Treat [Internet]. 2011;2011. Available from: http://www.ncbi.nlm.nih.gov/pmc/articles/PMC3123910/. Accessed 22 Mar 2016. 10.1155/2011/806298 PMC3123910 21738869

[60] Corrigan PW, Salzer M, Ralph RO, Sangster Y, Keck L (2004). Examining the factor structure of the Recovery Assessment Scale. Schizophr Bull.

[61] Rogers ES, Chamberlin J, Ellison ML, Crean T (1997). A consumer-constructed scale to measure empowerment among users of mental health services. Psychiatr Serv Wash DC.

[62] Thompson K, Kulkarni J, Sergejew AA (2000). Reliability and validity of a new Medication Adherence Rating Scale (MARS) for the psychoses. Schizophr Res.

[63] Bech P, Olsen LR, Kjoller M, Rasmussen NK (2003). Measuring well-being rather than the absence of distress symptoms: a comparison of the SF-36 Mental Health subscale and the WHO-Five Well-being Scale. Int J Methods Psychiatr Res.

[64] Meyer TJ, Miller ML, Metzger RL, Borkovec TD (1990). Development and validation of the Penn State Worry Questionnaire. Behav Res Ther.

[65] Kessing LV, Hansen HV, Ruggeri M, Bech P (2006). Satisfaction with treatment among patients with depressive and bipolar disorders. Soc Psychiatry Psychiatr Epidemiol.

[66] Hansen HV, Christensen EM, Dam H, Gluud C, Wetterslev J, Kessing LV. The effects of centralised and specialised intervention in the early course of severe unipolar depressive disorder: a randomised clinical trial. PloS One. 2012;7(3):e32950. doi:10.1371/journal.pone.0032950. 10.1371/journal.pone.0032950 PMC3307703 22442673

[67] Schulz KF, Grimes DA (2002). Allocation concealment in randomised trials: defending against deciphering. Lancet.

[68] Schulz KF, Grimes DA (2002). Unequal group sizes in randomised trials: guarding against guessing. Lancet.

